# Expanding the coverage of spatial proteomics: a machine learning approach

**DOI:** 10.1093/bioinformatics/btae062

**Published:** 2024-02-03

**Authors:** Huangqingbo Sun, Jiayi Li, Robert F Murphy

**Affiliations:** Computational Biology Department, Carnegie Mellon University, Pittsburgh, PA 15213, United States; Computational Biology Department, Carnegie Mellon University, Pittsburgh, PA 15213, United States; Computational Biology Department, Carnegie Mellon University, Pittsburgh, PA 15213, United States

## Abstract

**Motivation:**

Multiplexed protein imaging methods use a chosen set of markers and provide valuable information about complex tissue structure and cellular heterogeneity. However, the number of markers that can be measured in the same tissue sample is inherently limited.

**Results:**

In this paper, we present an efficient method to choose a minimal predictive subset of markers that for the first time allows the prediction of full images for a much larger set of markers. We demonstrate that our approach also outperforms previous methods for predicting cell-level protein composition. Most importantly, we demonstrate that our approach can be used to select a marker set that enables prediction of a much larger set than could be measured concurrently.

**Availability and implementation:**

All code and intermediate results are available in a Reproducible Research Archive at https://github.com/murphygroup/CODEXPanelOptimization.

## 1 Introduction

Spatial proteomics methods enable researchers to analyze individual cell properties and their spatial relationships in complex tissues. They can be divided into methods that can detect all proteins expressed above a given level (such as imaging mass spectrometry) and methods that measure levels of a pre-chosen set of proteins [such as multiplexed methods like co-detection by indexing (CODEX) and imaging mass cytometry (IMC)] (Hickey *et al.* 2022). However, the latter methods allow only a small fraction of all proteins to be imaged in the same sample. Different methods differ in spatial resolution, marker sensitivity, and number of markers that can be detected.

Deep learning has been widely used to solve a variety of biomedical image analysis problems including cell segmentation (Czech *et al.* 2019, Zhao and Yin 2020, Cutler *et al.* 2022, Greenwald *et al.* 2022), super-resolution microscopy enhancement (Shah *et al.* 2021, Qiao *et al.* 2022), and cell type identification (Brbić *et al.* 2020, Hickey *et al.* 2021, Brbić *et al.* 2022). In particular, it helps to learn complex relationships between different signal sources from large collections of images. A particularly useful approach for imaging is “*in silico* labeling,” in which unmeasured signals are predicted from easily acquired common reference signals. It includes predicting fluorescent tags of subcellular components from unlabeled microscope images (Christiansen *et al.* 2018, Ounkomol *et al.* 2018, Wang *et al.* 2021, Sun *et al.* 2022), virtual histological staining of tissue images (Rivenson *et al.* 2019, Li *et al.* 2020, Zhang *et al.* 2020), and predicting immunofluorescence or directly inferring cell types from immunohistochemical stained images (Yuan *et al.* 2021, Ghahremani *et al.* 2022). This concept can be extended to predict a large number of protein markers from images of a smaller number (Ternes *et al.* 2022, Saurav *et al.* 2023). For example, Wu *et al.* (2023) described a method to select 7 protein markers out of 40 that enabled accurate prediction of cell types in a number of tissues, and showed the effectiveness of the approach by imaging only those 7.

**Figure 1.**
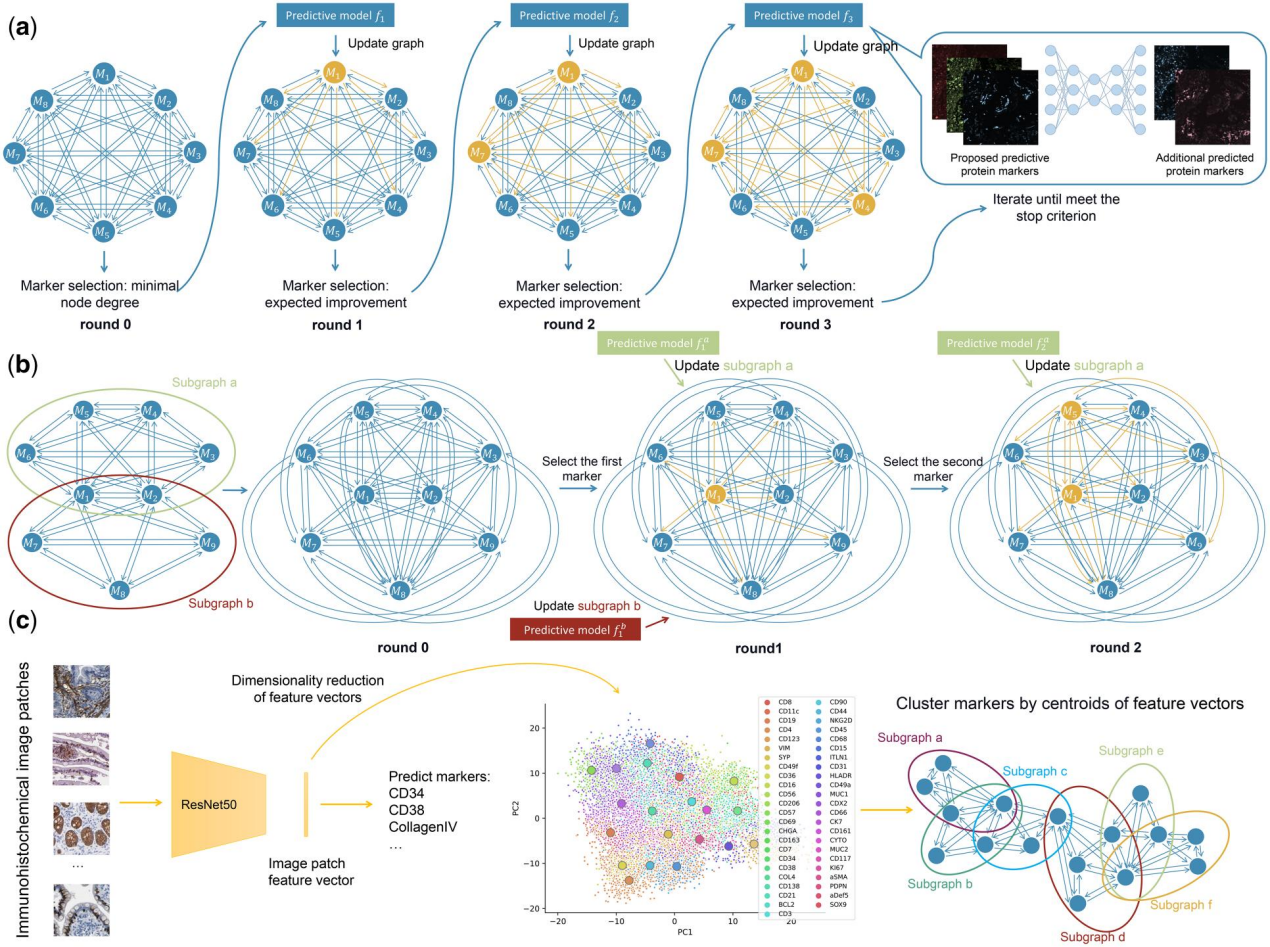
Overview of our methods for identifying predictive marker sets. (a) Single-panel setting. A clique is constructed with a node for each marker. Then, nodes are iteratively added to a predictive subset (shown in yellow) based on their expected improvement (see Section 2), a predictive model is trained from that subset (illustrated on the right), and edges attached to them are updated by the predictive model’s performance (also shown in yellow). (b) Multi-panel setting. The desired set of markers is partitioned into panels (two in this case) with some overlap ($\left\{M_{1},M_{2}\right\}$). Predictive models are then constructed from each panel separately. In Round 0, the graph is completed by assigning upperbound edge loadings to edges connecting markers in different panels using the triangle inequality. Markers are iteratively chosen using the graph to be added to the predictive set, and predictive models are retrained for one or more panels as appropriate. (c) Panel creation. Markers are partitioned into smaller panels based on the centroids of their associated feature vectors extracted with a trained immunohistochemical image classifier.

In this work, we first sought to develop a flexible approach for finding a small subset of markers and using them to predict the full-image expression pattern of the remaining markers. (Note that we use the generic term “ marker” here since, while this refers to a protein/antibody marker in the datasets we have used as examples, our methods can in principle be applied to any type of molecule detection approach involving a pre-chosen set.) In contrast to the method of Wu *et al.* (2023), we consider the problem of marker selection from an optimization standpoint; our single-panel setting focuses on selecting markers by explicitly modeling the spatial relationship between marker expression levels/intensities with a graphical model and using a neural network to directly predict the full marker image instead of predicting expression at the single-cell level. Our basic approach is illustrated in Fig. 1a.

In addition, we consider a more difficult setting in which all markers of interest cannot be imaged simultaneously and therefore deep learning models cannot be trained for all of them. We show that our proposed method can effectively address this problem (Fig. 1b and c), and the method can be easily extended to a very large number of markers of interest.

## 2 Materials and methods

### 2.1 *Data-driven* protein marker panel design

First, we introduce the problem and an overview of our proposed heuristic approach. Formally, we denote markers of interest as a set of random variables (RVs) $X=\{X_{1},\ldots ,X_{N}\}$, and our goal is to partition X into $X_{\text{obs}}$ and $X_{\text{pred}}\subseteq X\backslash X_{\text{obs}}$, where $X_{\text{obs}}$ denotes the set of predictive markers and $X_{\text{pred}}$ denotes the set of markers to be predicted. We write the partition as δ: X→{0, 1}, let $\delta_{i}$ denote $\delta (X_{i})$. That is, $X_{\text{obs}}=\{X_{i}:\;\delta_{i}=1\}.$ Given a partition, the next step is to construct a predictive model $f\in F:\;X_{\text{obs}}\to X_{\text{pred}}$. In this work, we use empirical risk minimization (ERM) to optimize *f*. We denote $D_{\text{obs}}$ as the distributions over $X_{\text{obs}}$. With the choice of cost function used in the learning algorithm, *L*, we write the risk of our predictive model as $R=E_{(x,y)∼D_{\text{obs}}\times D_{\text{pred}}}L(f).$ Then, the problem of finding a minimal predictive set can be viewed as the following optimization programming,

(1) $$X_{\text{obs}}=\underset{A\in P(X)}{\text{argmin}}|A|\;\;s.t.\rho ,$$

where ρ denotes the chosen stopping criterion and P denotes the powerset.

In general, finding an exactly minimal set of markers is computationally hard.

### 2.2 Single-panel

Given the hardness of the problem, we used a heuristic algorithm. Our algorithm starts with constructing a directed graph G=(V, E) where nodes in V are associated with the RVs in X. We use nodes $v_{i}\in V$ and RV $X_{i}$ interchangeably in the following text. E denotes the edge set of G where $E=\{e_{i,j}=(X_{i},\;X_{j}):\;\forall i,\;j\in [|X|],\;i\neq j\}$, where each edge has a non-negative *loading* $w_{i,j}$. Assume we have some dissimilarity measure ξ over X×X, we then initialize the edge loading by the dissimilarity between the RVs associated with the nodes, i.e. the dissimilarity between the expression patterns of those two markers. In practice, we use L1-norm as the dissimilarity measure in this work. Assume we have a training set *S* and a validation set *P*. Initially, we set the loadings as follows,

(2) $$w_{i,j}={\hat{E}}_{S}\xi (X_{i},\;X_{j}),\;e_{i,j}\in E,$$

where ${\hat{E}}_{S}$ denotes the empirical expectation over *S*. After initializing G, we assign a *cost* to each node in V by a non-negative function q: V→R, where q(v) denotes the cost of node *v*, ∀v∈V. Also, we distinguish edges by if they are activated, an activated edge means it associates at least one node whose associated RV is in $X_{\text{obs}}$ and goes out from the node in $X_{\text{obs}}$. When $e_{i,j}$ is activated, we denote $\kappa_{i,j}=1$. That is, $\kappa_{i,j}=1$ if $X_{i}\in X_{\text{obs}}$ and 0 otherwise. For a node whose associated RV in $X_{\text{pred}}$, the node cost is assigned by the minimal edge loading of activated edges it associates with, and 0 for a node whose associated RV is in $X_{\text{obs}}$. That is, $\forall X_{i}\in X$

(3) $$q(v_{i})=\{\begin{array}{cc} \underset{X_{j}}{\min}w_{j,i},\;\text{such that}\;\kappa_{j}=1 & X_{i}\in X_{\text{pred}}\\ 0 & X_{i}\in X_{\text{obs}}. \end{array}$$

We start with $X_{\text{in}}$ containing only one RV, whose associated node had the minimal node cost. Then, in each iteration, the predictive model $f^{t}$ is trained by ERM and predictions are made for RVs in $X_{\text{pred}}$. Our goal is to have node costs measuring the unpredictability of their associated RVs, and edge loadings measuring the risk occurring when predicting the value of one connected node from the other observed connected node, i.e. the dissimilarity between predicted and real patterns. We update the loadings by the predictive model’s generalization performance on the held-out validation set *P* after training a new predictive model, i.e. $\forall X_{i},X_{j}\in X,\;X_{i}\neq X_{j},$

(4) $$w_{i,j}=\{\begin{array}{cc} {\hat{E}}_{P}\xi ({\hat{X}}_{j}^{t},X_{j}) & X_{i}\in X_{\text{obs}},\;X_{j}\in X_{\text{pred}}\\ 0 & X_{i},X_{j}\in X_{\text{obs}}\\ w_{i,j} & \text{otherwise} \end{array}$$

where ${\hat{X}}_{j}^{t}$ denotes the prediction of $X_{j}$ from predictive model $f_{t}$. Note that the loading of an edge from a predicted node to an observed node will not be updated since the unpredictability is only regarding the direction from observation to prediction. In general, the initial dissimilarity measure between the expression patterns of a pair of markers is an approximate upper bound of the unpredictability (i.e. the value that would result if the prediction is trivially made by outputting the input patterns). The algorithm aims to gradually reduce the overall unpredictability among all markers of interest by iteratively including informative markers into $X_{\text{obs}}$, as illustrated in Algorithm 2. The principle of “expectedImprovement” is that we select an RV in $X_{\text{pred}}$ and add it to $X_{\text{obs}}$ in order to decrease the risk the most. Since the predictability of predictive models is unknown without further assumption, our method seeks to iteratively reduce the approximated upper bound of the unpredictability.

In practice, we selected the RV that would most decrease the summation of the node costs $\sum_{i\in [|X|]}q(v_{i})$. After selecting a new marker into the predictive set, we retrain the predictive model using the updated set of selected markers. We illustrated the full algorithm in Algorithm 1.

Algorithm 1 Predictive Marker Identification

**Require:** S, P, F, X, ρ

1: t←0

2: $X_{\text{obs}}\leftarrow \emptyset$

3: setup edge weights by eq. 2

4: $X_{\text{obs}}\leftarrow X_{\text{obs}}\cup {\text{argmin}}_{X_{i}}\text{deg}(v_{i})$▹ deg refers to node degree

5: $X_{\text{pred}}\leftarrow X\backslash X_{\text{obs}}$

6: **while** not ρ**do**

7:  t←t+1

8:  $f_{t}={\text{argmin}}_{f\in F}R_{S}$▹ train the model by ERM

9:  $\forall X_{i},X_{j}\in X,\;X_{i}\neq X_{j},$ update edge loadings by 4

10:  $\forall X_{i}\in X,$ update node costs by 3

11:  $X\leftarrow \mathrm{expectedImprovement}(X_{\text{obs}},\;X_{\text{pred}},\;W,\;\kappa )$

12:  $X_{\text{obs}}\leftarrow X_{\text{obs}}\cup X$

13: $X_{\text{pred}}\leftarrow X\backslash X_{\text{obs}}$

14: **end while**

15: **return**$X_{\text{obs}}$

Algorithm 2 subroutine: expectedImprovement

**Require:** $X_{\text{obs}},\;X_{\text{pred}},\;\{w_{i,j}:e_{i,j}\in E\},\;\{\kappa_{i,j}:e_{i,j}\in E\}$

1: $I=\{i:\;X_{i}\in X_{\text{out}}\}$

2: **for** each element k∈I**do**

3:  **for**i∈[|X|]**do**

4: $q^{\prime }(v_{i})\leftarrow \{\begin{array}{cc} \underset{X_{j}}{\min}w_{j,i},\;s.t.\;\kappa_{j,i}=1\;\text{or}\;j=k. & X_{i}\in X_{\text{pred}}\\ 0 & i=k\;\text{or}\;X_{i}\in X_{\text{in}} \end{array}$

5:  **end for**

6:  $\psi (k)\leftarrow \sum_{i=1}^{\left|X\right|}q^{\prime }(v_{i})$

7: **end for**

8: $p\leftarrow {\text{argmin}}_{k\in I}\psi (k)$

9: **return**$X_{p}$

### 2.3 Multi-panel

The single-panel setting assumes the training and validation sets consist of images with all markers of interest observed. However, this is not always true when the number of markers of interest is large. For example, suppose we have 200 protein markers of interest, but current imaging technology only allows us to have up to 60 protein markers in a single image. Then, the single-panel setting will fail as it requires the samples in the *S* and *P* to contain all RVs in X in order to initialize G. We, therefore, extended the algorithm to solve this problem.

For simplicity, we first consider the case that the markers of interest, X, can be measured using only two panels. We denote the markers in the two panels by $X_{A}$ and $X_{B}$ respectively, where $X_{A}\cup X_{B}=X.$ Here, we assume the sizes of both panels are less than or equal to the maximum number of markers that can be imaged simultaneously. Also, we expect there will be a set of *overlapping markers* appearing in both panels, namely $X_{M}=X_{A}\cap X_{B}\neq \emptyset .$ For markers in every single panel, we can first set up individual sub-graphs using the same approach as for the single-panel setting described above. We then consider completing the whole graph by inferring the loadings of edges between RVs in different panels. Recall that the edge loadings are initialized by the dissimilarity of two RVs and updated by their unpredictability from one to the other RV associated with their connecting nodes. If we assume the unpredictability is a valid norm (e.g. L1-norm), for any two markers not in the same panel, we can get an upper bound on the edge loading between $X_{i}$ and $X_{j}$ by the triangle inequality, $w_{ij}\leq w_{iq}\;+\;w_{qj}$, for $X_{q}\in X_{M}$. By doing so, the distributed version degenerates to the single-panel setting for which we already had an approximate solution above. Each round, two predictive models are retrained with respect to markers in $X_{A}$ and $X_{B}$ respectively using the training sets $S_{A}$ and $S_{B}$, depending on the panel where the last marker was selected. In the multi-panel setting, the loading of edge $e_{i,j}$ is initialized by the following three cases: when both $X_{i}$ and $X_{j}$ both in the overlap set $X_{M}$, the edge loading is the average dissimilarity (measured by ξ) between two associated RVs among two panels, as $w_{i,j}=\frac{1}{2}\left({\hat{E}}_{S^{A}}\xi (X_{i},X_{j})+{\hat{E}}_{S^{B}}\xi (X_{i},X_{j})\right)$; when two nodes are both in a single panel and not both in the overlap set, we have $w_{i,j}=E_{S^{A\;\text{or}\;B}}\xi (X_{i},X_{j})$; and lastly, when two nodes locate in separated panels, we apply the triangle inequality as $w_{ij}=\min_{X_{q}\in X_{M}}w_{iq}\;+\;w_{qj}$.

It is then trivial to extend the method to a multi-panel setting. That is, consider the whole set of markers X being partitioned into *m* subsets associated with *m* panels, i.e. $X=\overset{m}{\underset{i=1}{\cup }}X_{i}$; and for each subset, there exists at least one other subset, their intersection is not empty (as in the two-panel case they share some overlap markers), which indicates the whole graph of marker RVs has no disconnected compartments. By doing so, for every two markers not in the same panel, we can find at least one path in the graph connecting their associated RVs, and therefore we can complete the whole set of edge loadings by chaining the triangle inequality and find the minimal loading value if there exist multiple paths. In practice, finding paths is realized by standard depth-first search. If a marker is predicted by multiple predictive models, the predictions will be averaged.

### 2.4 Data preprocessing and machine learning

To illustrate the single-panel setting, we used images from two different proteomics methods. The first was spleen and lymph node CODEX image datasets from data published by the Snyder *et al.* (2019). These contained eight and nine multichannel images of different tissue regions respectively. We split the datasets into training, validation, and test sets of 4:2:2 for the spleen dataset and 4:2:3 for the lymph node dataset. Each image in those datasets contains 29 measured protein markers. (The details of image data used in this study are listed in Supplementary Table S1.) The second was a Human Tumor Atlas Network dataset containing 75 multichannel images of pancreaticobiliary-type carcinoma samples (Rozenblatt-Rosen *et al.* 2020); these were randomly partitioned into training, validation, and test sets (45:15:15). The images contain 27 or 28 channels for different protein markers, and we used 24 channels in our experiment (removing markers that either did not appear in all images or barely contained nontrivial patterns).

For testing the multi-panel settings, we used HuBMAP datasets for large and small intestine image datasets containing 16 different multichannel images, where each image contains 46 measured protein markers. For both datasets, four images were held out as a separate test set. The remaining 12 images were evenly split into training and validation sets.

The intensities of each channel were normalized for each CODEX image using the same normalization method as Wu *et al.* (2023) for spleen and lymph node datasets, as follows

(5) $$x_{i}=z-\text{score}\left(\text{arcsinh}\left(\frac{x_{i}}{\max(5q_{0.2}(x_{i}),15)}\right)\right),$$

where $x_{i}$ denotes the *i*th channel of the image, $q_{0.2}$ denotes the 20th percentile, and $z-\text{score}(x):=\frac{x\;-\;\mu_{x}}{\sigma_{x}}$ given $\mu_{x}$, $\sigma_{x}$ denotes the mean and standard deviation of *x* respectively. For pancreas data, each image, for each channel, was clipped to the range of 0 to the 98 percentile of all positive pixel values, then blurred using a Gaussian kernel (with a sigma of 1.5), and *z*-score normalized. For small and large intestine datasets, we directly applied *z*-score normalization, since the signal in these two datasets is relatively weak and sparse. The normalized image data were used to train the machine learning models.

We used convolutional neural networks as the predictive model in this work. In particular, the network was a U-Net (Ronneberger *et al.* 2015) with skip connections (He *et al.* 2016) which has been widely used in modern computer vision applications (the network architecture is shown in Supplementary Fig. S1). The network was used as an end-to-end predictive model, whose input was the image patch containing channels associated with $X_{\text{obs}}$ and whose output was the image channels associated with $X_{\text{pred}}$. The predictive model was trained to minimize the empirical mean square error (MSE) using an Adam optimizer with a learning rate of $10^{-4}$. Since the size of each whole-slide CODEX image is very large, during training time, randomly cropped image patches from the whole-slide CODEX image with the size of #channel×192×192 were used. Specifically, each cropped image patch was split into two images, one with channels associated with $X_{\text{obs}}$, and the other with channels associated with $X_{\text{pred}}$); the resulting two images were used as the input and target for the model training. During the validation or testing time, the patches were cropped as a sliding window (not randomly) from a whole-slide CODEX image containing only channels in $X_{\text{obs}}$, and the whole-slide image was recovered by stitching together predictive and predicted patches. Note that the patch size (#channel×192×192) in this work was chosen to fit the U-Net architecture so that it can produce the same image patch size after the forward propagation (the architecture can be adjusted for other patch sizes). The validation set was used to monitor the training process and the model with the lowest validation loss was selected.

The running time for training a model from scratch for a single iteration on a single GPU was ∼4 h; however, this of course may vary for different computing resources and dataset sizes. As the predictive set selection required re-training models each round (Line 8 in Algorithm 1), to reduce the running time, we let the model in round t+1, $f_{t+1}$ inherit the trained weights from round *t*, $f_{t}$, for t>0. Since the network architecture of $f_{t+1}$ had an additional input channel and one fewer output channel compared to $f_{t}$, the weights of the selected channel in the input layer were randomly initialized in $f_{t+1}$ and the weights of this channel in the output layer of $f_{t}$ were not inherited. We randomly initialized the weights of $f_{1}$ following a normal distribution.

### 2.5 Quality measures

Our first assessment is the overall unpredictability of X, i.e. the sum of node costs, measured by the L1-norm. A second is the predictive model’s performance on the test set in terms of reconstruction MSE and Pearson Correlation Coefficient (PCC) between synthetic and real images.

We also examined the quality of single-cell level predictions. We first segmented individual cells from test images. For spleen and lymph node datasets, we directly used segmented masks from HuBMAP. For intestine datasets, we segmented the cells using DeepCell Mesmer (Greenwald *et al.* 2022). We then created matrices (for both real and synthetic images) whose rows refer to individual cells, columns refer to the protein markers, and each entry refers to the average expression intensity of a protein marker for a particular cell. We also created another single-cell profile referred to as the correlation profile, whose entries are the PCC between the expression patterns of a pair of protein markers within a cell region. That is, the columns of this matrix refer to every pair of markers of interest. We measured the difference between the matrices resulting from real and synthetic images using the normalized Frobenius norm.

### 2.6 Panel formation using immunohistochemical images

For the multi-panel study, we designed the sub-panels according to the similarity of the expression patterns of protein markers. Since predictive models will be trained for each sub-panel separately, similarities within a panel give the best chance of their being predictable from each other. The similarity was measured by feature vectors resulting from a neural network classifier trained on immunohistochemical image patches. For this we used 334 and 336 whole-side images of 41 protein markers available for colon and small intestine from the Human Protein Atlas (Uhlén *et al.* 2015) (for markers without immunohistochemical images, we randomly added them into sub-panels). Here, for each whole slide immunohistochemical image, we decomposed it into two channels referring to the protein marker and tissue background using the algorithm described in Newberg and Murphy (2008), and randomly cropped it into 3×224×224 patches from regions with high protein expression (the size of patch is chosen to fit a pre-trained image classifier). For each tissue, we randomly split all patches into training and validation sets and trained a ResNet-50 based image classifier to learn to classify protein expression patterns according to their associated protein markers. To train this image classifier, we started from a model pre-trained on the ImageNet, and then fine-tuned it for 150 epochs on our immunohistochemical image datasets (for every 30 epochs, we saved a checkpoint model). The feature vector associated with each image patch was the average internal embedding generated by all checkpointed models. That is, for each image, we extracted the embeddings (the output from the second-to-last layers) from all checkpointed models, and then averaged them as a single feature vector. We then collected all these feature vectors associated with each image patch (from both training and validation sets) and performed PCA to reduce their dimensionality to 2. Next, we grouped the protein markers into five clusters manually according to centroids of their feature vectors in the reduced PC coordinates (see Supplementary Fig. S5a and c). Then, the initial panels were manually adjusted and extended while balancing the number of markers in each panel and ensuring overlap between panels (Supplementary Fig. S5b and d).

## 3 Results

### 3.1 Single-panel experiment

#### 3.1.1 CODEX imaging

**Figure 2.**
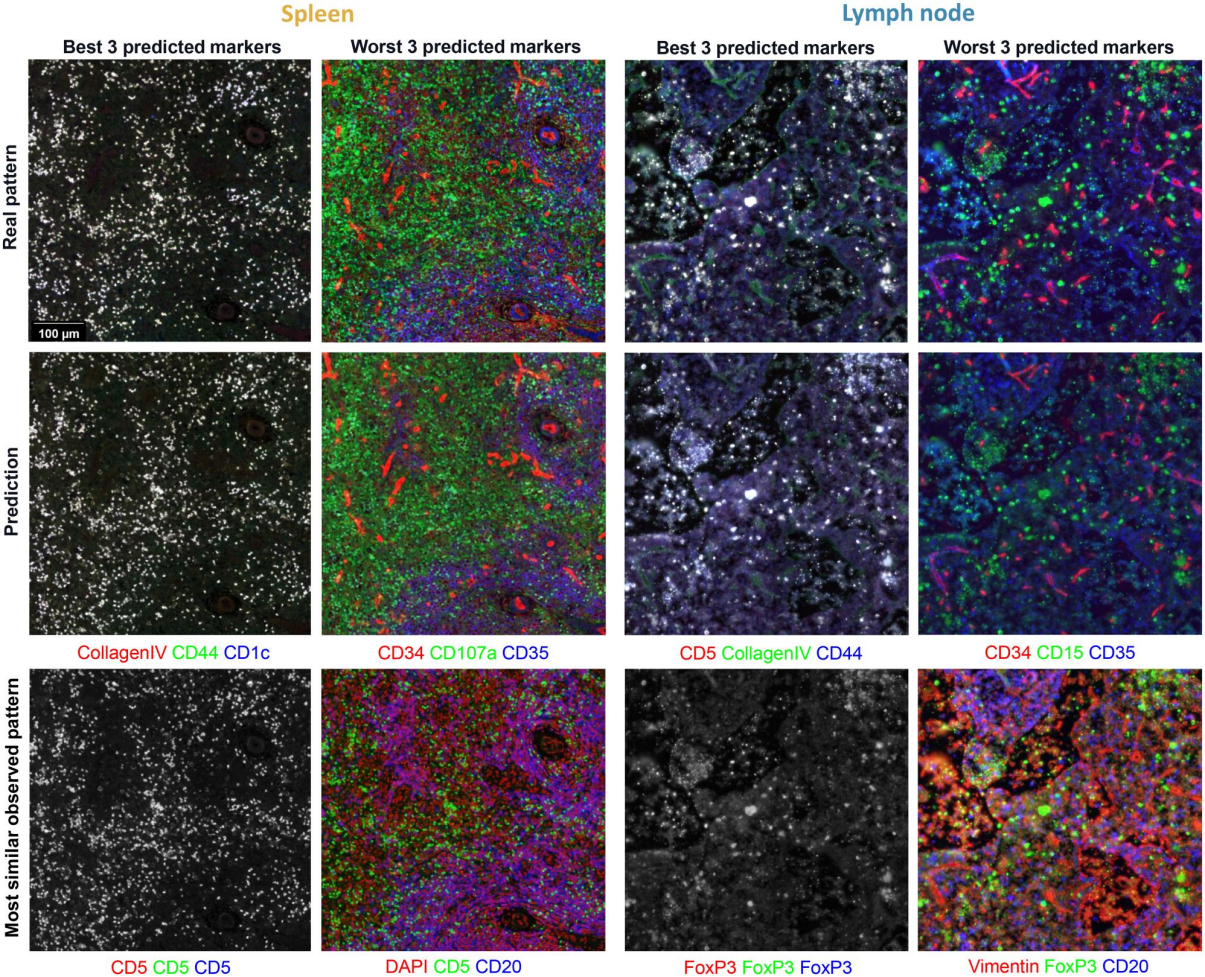
Comparison of predictions of protein marker expression. Example patches from synthetic and real test images for the best and worst three predicted protein markers for spleen and lymph node are shown in the first two rows. (Images in some panels barely show any color due to the three expression patterns being similar or identical.) The expression patterns of the protein markers from the selected predictive set that are most similar to the illustrated predicted protein markers are shown in the third row. For example, for the worst predicted protein markers in spleen (second column), DAPI was most similar to CD34, CD5 to CD107a, and CD20 to CD35. Note that accurate predictions can be made even for markers whose patterns are visibly different from the input patterns (e.g. CD34).

**Figure 3.**
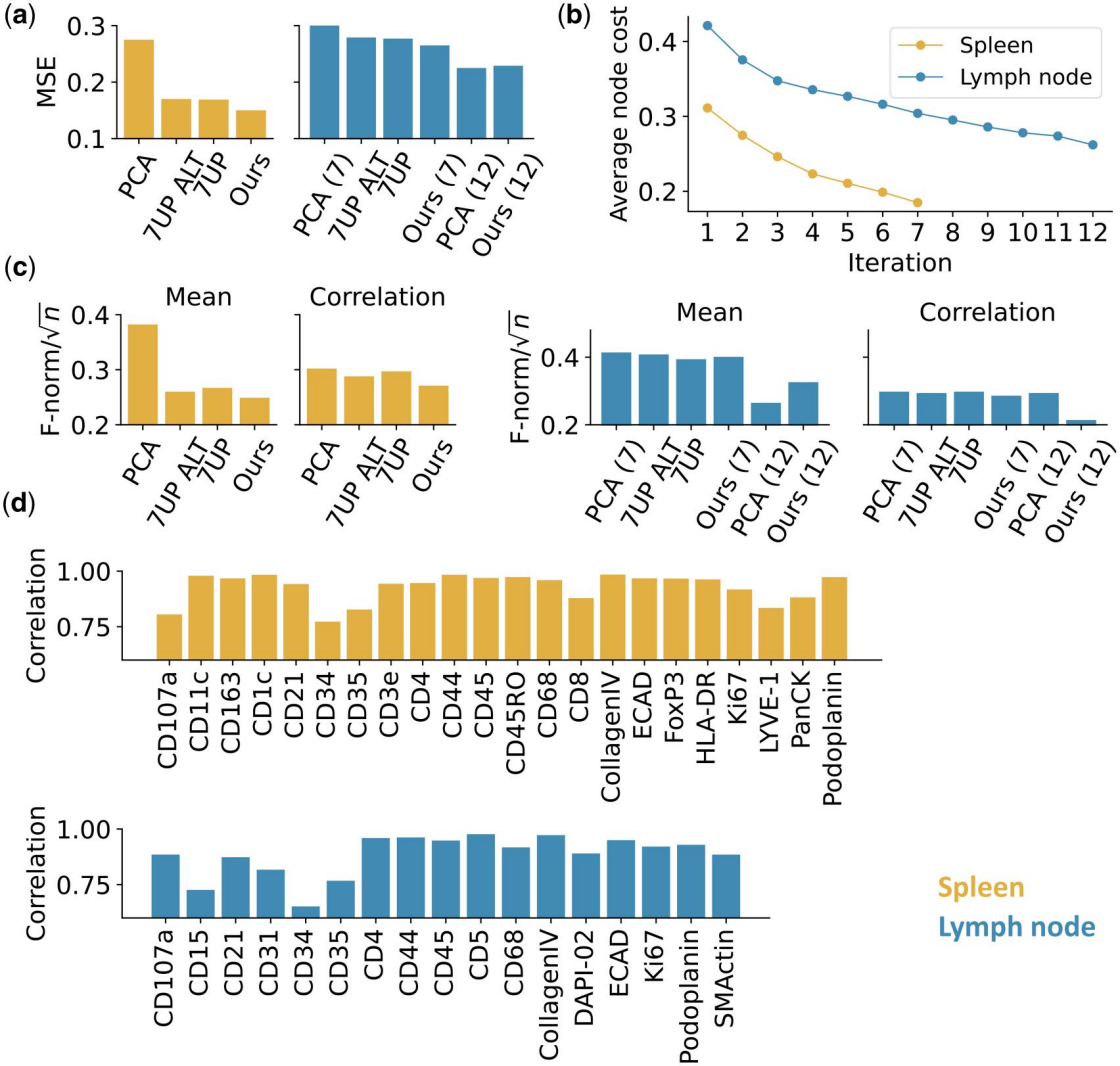
Evaluation of prediction approaches. (a) Reconstruction error (MSE) for various prediction approaches. PCA refers to simply selecting a predictive set using the markers with the most variance in intensity. For lymph node, the number inside the parentheses indicates the number of protein markers selected. (b) The changes of the average node cost (the overall unpredictability to be minimized) throughout the marker selection procedure. (c) Single-cell level assessment (lower values are better). (d) The pixel-wise PCC between synthetic and real images for each predicted protein marker.

To evaluate our approach, we used spleen and lymph node CODEX image datasets from the Snyder *et al.* (2019). As described in Section 2, each image was first normalized and randomly cropped into small patches. Given a subset of markers, predictive models were trained by minimizing the mean square error (MSE) between the predicted and observed expression intensities of the markers. Visual examination of examples of real and predicted image patches (Fig. 2) for the markers that are predicted the best reveals their patterns are essentially indistinguishable, and that those with the worst predictions are still quite similar. We numerically compared predictions using our selected sets with those of the sets described by Wu *et al.* (2023) and found that our method yields smaller differences between predicted and real images for both tissues (Fig. 3a). (For context, since channel intensities were normalized to z-scores, the expected MSE for random predictions within a range of 2 SD is ∼1.15; since it is calculated over thousands of pixels the chance of obtaining MSE values below 0.3 at random is infinitesimal.) By visually examining the most similar expression patterns from the input to those well-predicted protein markers, the predicted patterns still have subtle differences from the input patterns. This indicates that predictive models indeed learn the protein expression pattern instead of simply producing a pattern similar to their input; as expected, the complexity of the pattern impacts its predictability. We observed that the errors for the lymph node images were larger than for the spleen when using only seven input channels [the number chosen by Wu *et al.* (2023)]. Principal component analysis (PCA) (Supplementary Fig. S2a) showed that the lymph node profile has a longer tail in its channel eigen-spectrum indicating greater interchannel variability. From an information theory standpoint, this means that more predictors (e.g. PCs) would be needed to obtain the same quality of prediction as for spleen. We therefore selected five additional predictive protein markers for that tissue; the improvement at each step in the training processes is shown in Fig. 3b and the edge loadings associated with the final graphs are shown in Supplementary Fig. S3. We also characterized the predictions for individual cells (via differences in average channel intensities or in correlations between intensities of pairs of channels) (Fig. 3c). Overall, our method achieved better performance in reconstructing single-cell profiles for both tissues than both versions of 7UP. In addition to these overall measures of reconstruction, we evaluated performance at predictions of individual protein markers (Fig. 3d). Excellent correlations between real and predicted intensity values were observed for almost all markers. Predictions for CD34 were the lowest for both tissues; presumably it would have been the next marker chosen to move to the predictive subset.

#### 3.1.2 Imaging mass cytometry

To test our method on images from a different imaging modality, we performed a single-panel experiment on a pancreas IMC dataset. The images have 27 or 28 protein expression channels, with 24 channels used in our experiment. The IMC images were clipped, Gaussian blurred and normalized (as described in Section 2). Similar to the previous experiment, the full-view images were randomly cropped into small patches after preprocessing and used to train the predictive models.

**Figure 4.**
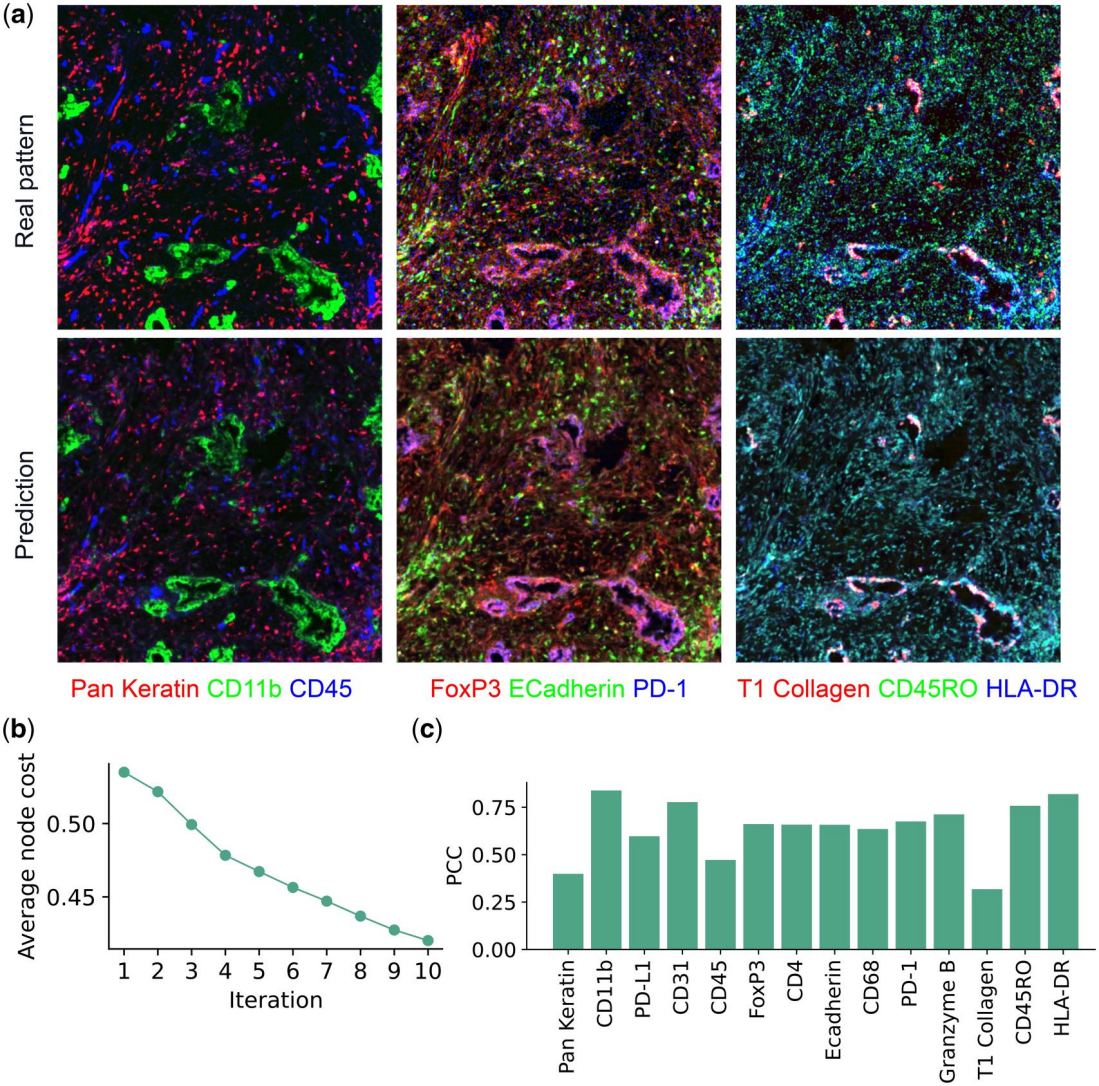
Results of single-panel method on IMC data. (a) Comparison of predictions of protein marker expression. Example patches from synthetic and real test images with representative protein markers for the pancreas IMC data are shown. (b) The changes of the average node cost throughout the marker selection procedure. (c) The pixel-wise PCC was measured between synthetic and real images for individual markers.

We iteratively selected 10 out of 24 markers for inclusion in the predictive set (Supplementary Table S3). As shown in Fig. 4a, visual examinations of examples of real and predicted image patches show that the predictive model can accurately predict most of the patterns, with the exception of the punctate patterns of Pan Keratin and T1 Collagen. This is also reflected in the estimates of the quality of reconstructions of individual protein markers shown in Fig. 4c. These proteins might have been expected to be chosen for inclusion in the predictive set, but since the selection process seeks to optimize the overall performance as reflected in node costs, it does not necessarily optimize each individual marker. As expected, the average node cost continuously decreases during the marker selection procedure (Fig. 4b). We note the average node costs in this experiment are higher than those for the spleen and lymph node datasets; this may be due in part to the relatively high spatial resolution and signal quality of CODEX images (making them more predictable) and in part to the differences in the protein sets. Nonetheless, the average MSE after 10 rounds is 0.578, which is lower than expected at random.

### 3.2 Multi-panel experiment

As discussed earlier, the number of protein markers that can currently be imaged in the same sample using multiplexed methods is in the dozens, while mammalian cells express tens of thousands of different proteins. Thus, creating a predictive set cannot be done by first collecting an image of all (or even hundreds of) markers. We therefore explored a more complicated setting for choosing a predictive set using images of different samples of the same tissue labeled with different panels of markers (see Fig. 1b). To test this multi-panel approach, we used small and large intestine CODEX datasets with 46 markers and simulated how well our algorithm would work if we could only image 19 markers per sample (i.e. by creating non-overlapping 19 channel images for each panel from the original CODEX images). For each dataset, we first made a holdout test set, then the remaining images were partitioned into training and validation sets (see Section 2). To benchmark performance, we ran the single-panel method on all 46 markers as an empirical upper bound for the multi-panel method.

**Figure 5.**
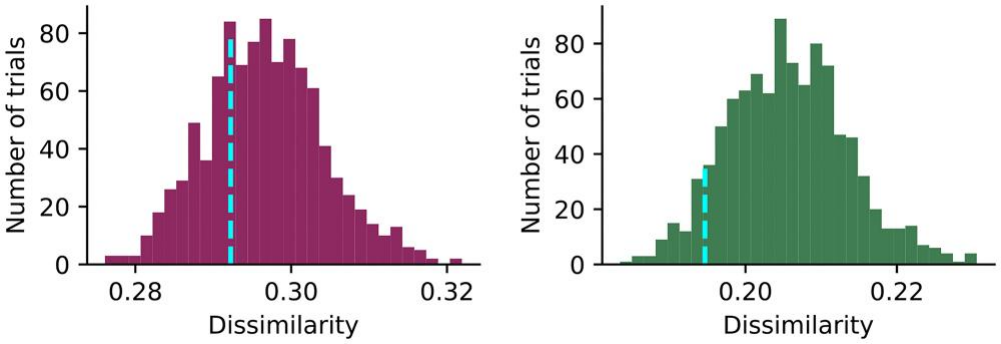
Evaluation of panel choices. The distributions of average dissimilarity between expression patterns of pairs of protein markers within each randomly partitioned panel are shown (Left: large intestine; right: small intestine). The average dissimilarity within panels partitioned using immunohistochemical images is also shown (dashline).

**Figure 6.**
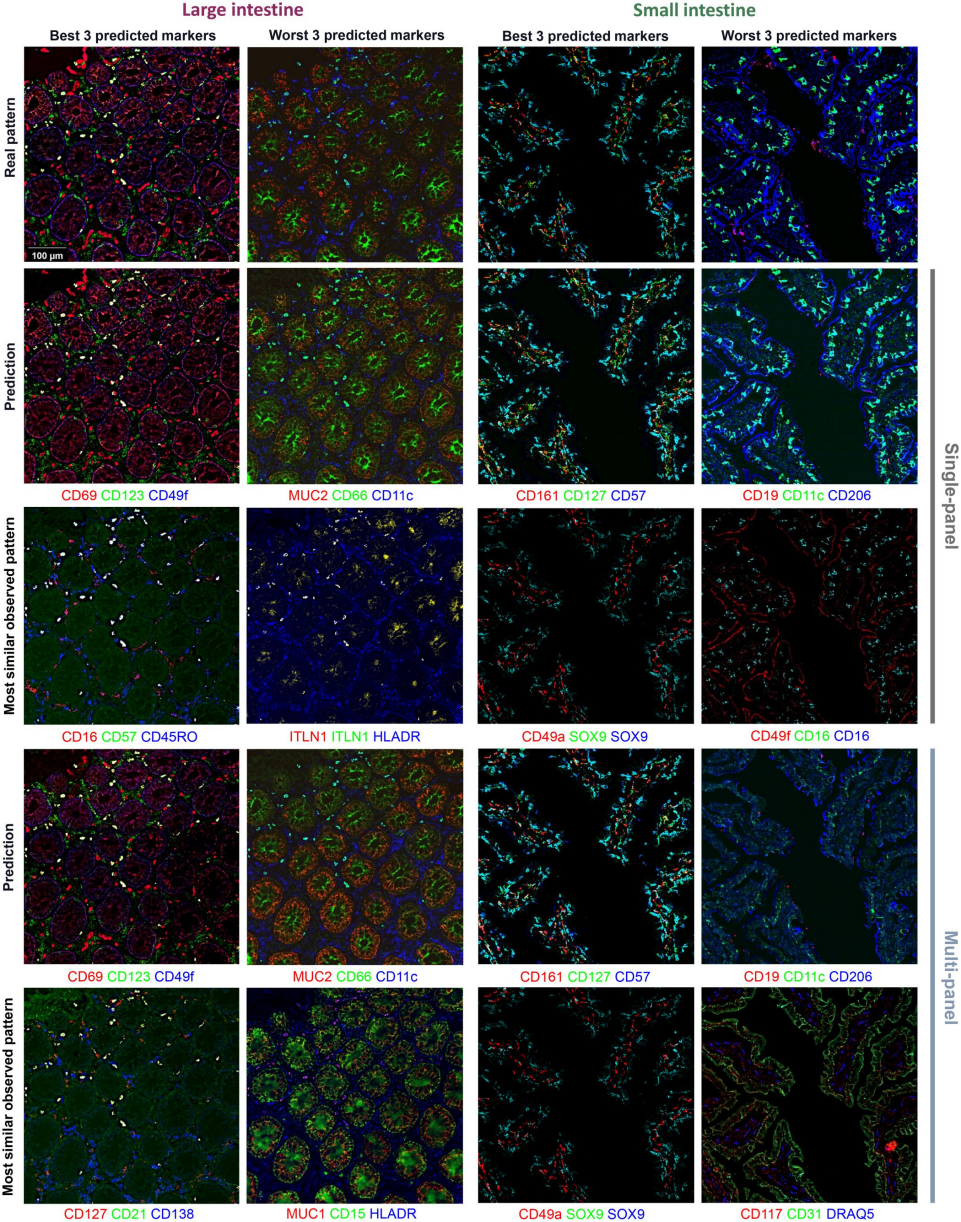
Comparison of protein marker predictions. Example patches from synthetic and real test images for the best and worst three predicted protein markers for the large and small intestine. The best and worst three protein markers were chosen from the intersection of predicted markers for the single and multi-panel settings. Similar to Fig. 2, the input expression patterns most similar to the corresponding real and predicted patterns are also shown.

**Figure 7.**
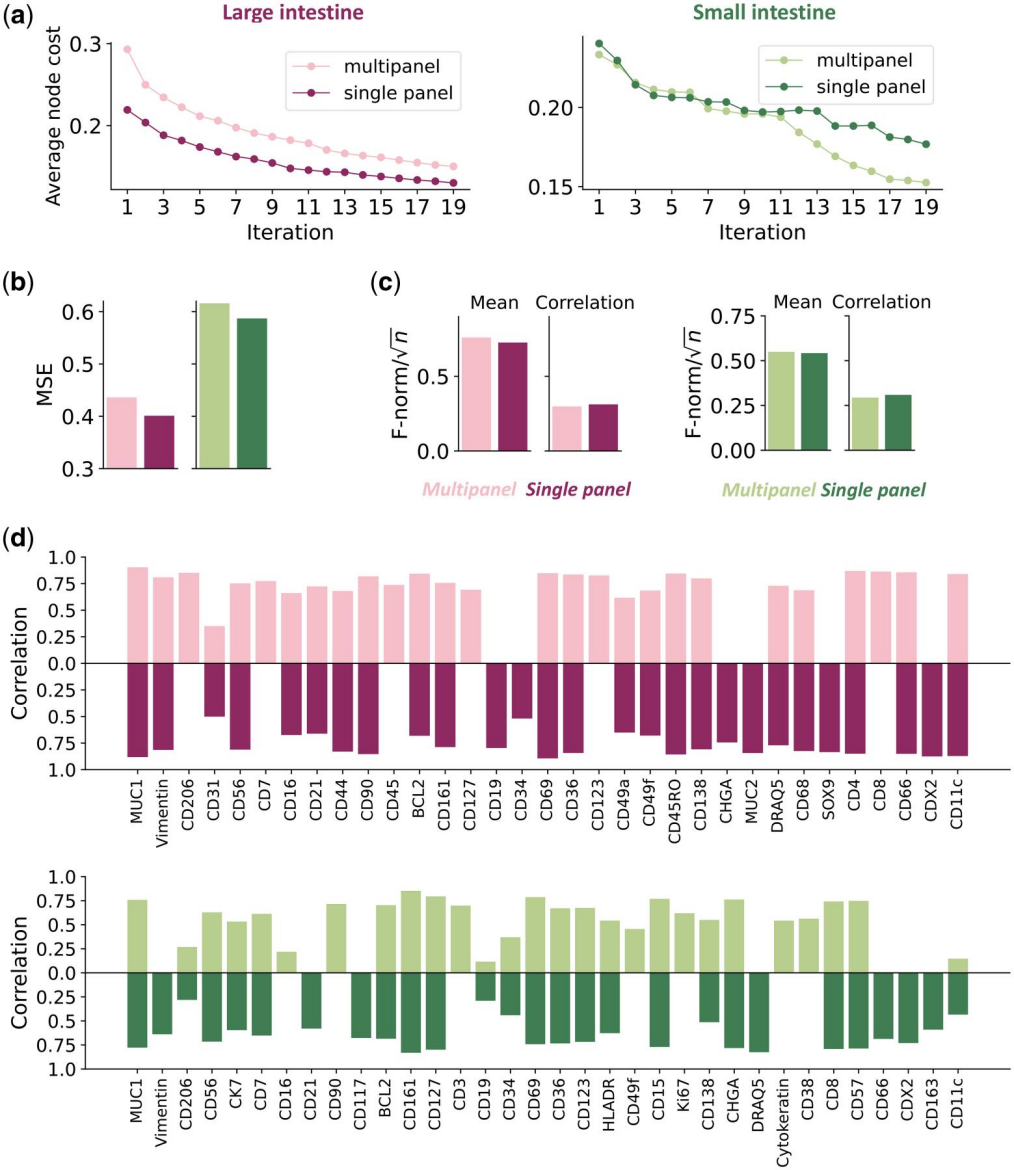
Evaluation of multi-panel prediction. (a) The changes of the average node cost (the overall unpredictability to be minimized) throughout the marker selection procedure. (b) Reconstruction error for various prediction approaches. (c) Single-cell level assessment (lower values are better). (d) The pixel-wise PCC between synthetic and real images for each protein marker. Bar colors indicate tissue and single/multipanel. Missing bars indicate that the corresponding proteins were in the predictive set for that model.

The first step in this multi-panel setting is to choose how to divide a desired large set of markers into smaller panels. Ideally, each panel would contain proteins that are similar to each other to maximize the chances of learning to predict them from each other. However, we do not know before imaging the panels what similarity the proteins would have in a given tissue sample. To provide an estimate of this, we used information extracted from independently acquired images from the Human Protein Atlas (Fig. 1c). All protein markers of interest were partitioned into five panels using similarities in immunohistochemical patterns (with some overlap; the panel design and composition are shown in Supplementary Fig. S5 and Supplementary Table S2). We then asked how well the similarities estimated using external immunohistochemical images predicted those observed in the CODEX images, compared to what would have occurred if the panels had been chosen randomly (Fig. 5). The results show that the panels for large intestine are less dissimilar (more similar) than most of the panels we would have obtained by random partitioning, and that they are quite a bit less dissimilar for small intestine (note that, since the panels include overlap, we cannot expect them to be completely dissimilar). We conclude that using external estimates for similarity is useful when we cannot directly estimate it. Once channels were chosen for each panel, we trained sets of models as shown in Fig. 1b. We show comparisons of best and worst predictions with their corresponding real images in Fig. 6. When the real and predicted patches are examined visually, both single- and multi-panel predictive models do well even for the worst predicted protein markers (with the exception of the multi-panel predictions for small intestine; the intensities are much lower, but the spatial distributions are still correctly predicted). The markers selected by either the single- or multi-panel approach are listed in Supplementary Table S3. For both datasets, a large proportion of protein markers are selected in both cases (12 and 13 out of 19). Consistent with this, the objective function declines slowly for both tissues and settings (Fig. 7a). The edge loadings of the final graphs under single- and multi-panel settings are shown in Supplementary Fig. S6. As shown in Fig. 7b, the difference in test MSE between the two settings is small. The prediction task for both intestine datasets appears to be harder compared to the spleen and lymph node datasets. Supplementary Figure S2b shows the fraction of explained variance for each principal component for small and large intestine cells; the protein expressions of small intestine cells are the most difficult to predict as the first PC explains <44% of variance (it also includes more markers with larger loadings, data not shown). For small and large intestine datasets, the first 10 PCs explain 75.26% and 84.27% of the variance, respectively, while they explain 96.32% and 90.71% of the variance in the spleen and lymph node datasets. To explore this, we examined the differences between the two settings at the single-cell level, and very similar performances between single and multi-panel are seen (Fig. 7c). Similar to the results in Fig. 3d, the correlation between pixel intensities in synthetic and real images were high for most individual protein markers under both settings (Fig. 7d). Note that protein markers associated with no bins mean they are not in the predicted marker set under either single- or multi-panel settings.

Lastly, we trained new predictive models on datasets from a different batch of experiments using our selected protein markers to see the generalization performance of our marker choices. We used the 19 protein markers to train the new predictive models on the latest published CODEX image sets of large and small intestine. Note that the new images have 54 protein markers, more than the number used in the marker selection. Two new predictive models were trained using the new datasets, and their generalization performances quantified by the test MSE were 0.591 and 0.536 (single-panel and multi-panel) for the new large intestine dataset and 0.563 and 0.583 (single-panel and multi-panel) for the new small intestine dataset respectively (roughly similar to our previous results even though the new datasets included eight previously unseen markers). These results indicate good generalization of the selected sets to new images.

## 4 Discussion

Despite recent advances in multiplexed imaging techniques, the number of protein markers that can be imaged in the same tissue sample is still limited. Based upon the expectation that some protein expression intensities can be predicted from other measured protein marker intensities, we have demonstrated an effective approach for making accurate predictions at the individual pixel level that outperforms a previous approach for the related problem of predicting cell-level intensities. From a deep generative modeling perspective, our approach can be potentially improved by incorporating different learning paradigms or using alternative deep learning models with richer expressiveness. For example, generative adversarial network or denoising diffusion based models (Goodfellow *et al.* 2020, Ho *et al.* 2020) could be used to improve predictive models. Note that the choice of predictive models is independent from the marker selection procedure we describe here. We have also shown that our method can be extended to learning an informative predictive subset even when the full desired set cannot be imaged in the same sample. This suggests the feasibility of constructing spatial networks for all proteins without imaging them in the same sample, and then synthesizing multiplexed protein images with high quality. While the number of proteins is of order $10^{4}$, our method potentially allows reduce the complexity of both the imaging and predictive model learning tasks since even if imaging of on the order of hundreds of panels is required, training models for each panel and estimating the edge loadings remain below order $10^{2}$. However, unlike the upfront partitioning of the graph into panels used here, an iterative approach to choosing and imaging panels may potentially be needed, with uncertain complexity. Future directions for optimizing protein marker panels include integrating other types of proteomics or genomics information into the graph.

In any case, expansion of the number of markers that can be analyzed in multiplexed imaging methods is expected to shed further light on protein and cell type spatial interactions in complex tissues.

## Supplementary Material

btae062_Supplementary_Data

## Data Availability

The data underlying this article are available in the HuBMAP repository at https://portal.hubmapconsortium.org/ and the Human Tumor Atlas Network WUSTL atlas (https://humantumoratlas.org), and can be accessed with unique identifiers as described in https://github.com/murphygroup/CODEXPanelOptimization.
