# Supplementary material for: Expanding the coverage of spatial proteomics: a machine learning approach
Source: Bioinformatics. 2024 Feb 3;40(2):btae062. doi: 10.1093/bioinformatics/btae062 (PMC10873576; doi:10.1093/bioinformatics/btae062)
Supplement: btae062_Supplementary_Data [file btae062_supplementary_data.pdf]

Expanding the coverage of spatial proteomics: a machine learning approach: Supplementary Information

Huangqingbo Sun, Jiayi Li, and Robert F Murphy

January 15, 2024

**Spleen**			
Training			
HBM495.VWBD.428	HBM825.KFFT.669	HBM396.BXSQ.568	HBM863.FDNH.844
Validation			
HBM455.PWQW.883	HBM967.TGDD.996		
Test			
HBM455.XDQS.993	HBM455.XDQS.993		
**Lymph node**			
Training			
HBM622.JXWQ.554	HBM992.RHJW.288	HBM268.NKXB.243	HBM557.SGTC.262
Validation			
HBM938.TNNT.879	HBM522.BSZT.385		
Test			
HBM347.PSLC.425	HBM997.PVCF.629	HBM685.TBGN.663	
**Large intestine**			
Training			
HBM334.QWFV.953	HBM353.NZVQ.793	HBM424.STVV.842	HBM462.JKCN.863
HBM622.STKS.394	HBM575.THQM.284		
Validation			
HBM683.NRPR.962	HBM729.XTBN.693	HBM739.HCWP.359	HBM429.LLRT.546
HBM438.JXJW.249	HBM439.WJDV.974		
Test			
HBM742.NHHQ.357	HBM792.FFJT.499	HBM938.KMNW.825	HBM964.FPNH.767
**New batch: large intestine**			
Training			
HBM396.FNQW.543	HBM423.MMGW.744	HBM352.MDZF.598	HBM292.FCMS.497
HBM953.LMWQ.235	HBM725.QFKT.594		
Validation			
HBM685.PCCJ.427	HBM753.VDXD.934	HBM634.MSKL.575	HBM994.KDNT.678
Test			
HBM946.NWTV.278	HBM524.VWGB.378	HBM886.NTZN.682	HBM494.VNTQ.422
**Small intestine**			
Training			
HBM443.LGZK.435	HBM466.XSKL.867	HBM666.RBCG.529	HBM284.SBPR.357
HBM676.QVGZ.455	HBM785.FJVT.469		
Validation			
HBM687.SJLD.889	HBM934.KLGL.584	HBM893.MCGS.487	HBM845.VMSZ.536
HBM899.KTQM.246	HBM945.FSHR.864		
Test			
HBM394.VSKR.883	HBM727.DMKG.675	HBM953.KMTG.758	HBM996.MDQH.988
**New batch: small intestine**			
Training			
HBM443.XPDK.549	HBM795.GWKV.825	HBM475.DXDC.532	HBM644.QKZS.857
HBM334.RPTP.997	HBM233.GTZN.466		
Validation			
HBM423.QJJR.545	HBM398.SWKV.256	HBM837.RHNC.533	
Test			
HBM587.VTDD.789	HBM829.QZQP.626	HBM735.PSTF.274	HBM962.BFPH.344

Table S1: HuBMAP CODEX image datasets used in this study.

**Large intestine**							
Panel 1	Hoechst	DRAQ5	CD127	CD4	CD44	CD49f	CD49a
	CD123	CD19	aSMA	CD66	CD138	CK7	ITLN1
	Cytokeratin	MUC1	MUC2	CD45	CD45RO		
Panel 2	Hoechst	DRAQ5	CD127	CD4	CD44	CD49f	CD49a
	CD45	CD45RO	CD57	CD69	CD117	Ki67	CD7
	CD8	CD11c	HLADR				
Panel 3	Hoechst	CD66	ITLN1	CD138	CK7	Cytokeratin	MUC1
	SOX9	Vimentin	CD38	CD31	CD36	BCL2	CHGA
	CD3	CD34	CDX2	CD161	CD15		
Panel 4	Hoechst	SOX9	Vimentin	CD38	CD31	CD36	BCL2
	CHGA	CD3	CD34	CDX2	CD161	CD15	CD206
	CD21	CD56	CD68	CD16	CD163		
Panel 5	Hoechst	DRAQ5	CD127	CD206	CD21	CD56	CD68
	CD16	CD163	CD90	CD57	CD69	CD117	Ki67
	CD7	CD8	CD11c	HLADR			
**Small intestine**							
Panel 1	Hoechst	DRAQ5	CD49f	CK7	CD19	CD123	CD66
	MUC1	CD4	CD138	CD44	ITLN1	CDX2	CD45
	CD45RO	CD117	MUC2	aSMA	CD31		
Panel 2	Hoechst	DRAQ5	CD57	CD90	MUC1	CD4	CD138
	CD44	CD15	CD11c	Ki67	CD69	CDX2	CD45
	CD45RO	CD117	ITLN1	CD49a	SOX9		
Panel 3	Hoechst	DRAQ5	CD15	CD11c	Ki67	CD69	CDX2
	CD45	CD45RO	CD117	CD49a	SOX9	CD68	CD21
	CD8	CHGA	CD161	CD7	CD56		
Panel 4	Hoechst	DRAQ5	CD127	CD49a	SOX9	CD8	CHGA
	CD161	CD7	CD56	BCL2	ITLN1	MUC2	aSMA
	CD31	Cytokeratin	CD36	CD3	CD38		
Panel 5	Hoechst	DRAQ5	CD127	CD206	HLADR	CD16	CD163
	Vimentin	CD34	CD68	CD21	CD8	CHGA	CD161
	CD7	CD56	CD49a	SOX9	BCL2		

Table S2: Multi-panel partitions used in this study derived from clustering of the features of immunohistochemical images. Note that Hoechst, DRAQ5, and CD127 markers (shown in gray) were not selected from the clustering results but rather manually added into panels as room was available.

**Spleen**						
CD5	DAPI	SMActin	CD31	CD15	Vimentin	CD20
**Lymph node**						
FoxP3	CD3e	PanCK	CD163	HLADR	Vimentin	CD8
CD20	CD1c	LYVE-1	CD11c	CD45RO		
**Pancreas**						
Ki67	Vimentin	Histone H3	Lag3	CD163	CD3	CD20
CD8a	CD45RA	CD11c				
**Large intestine**						
Single panel						
CD16	CD49a	Hoechst	aSMA	Vimentin	CD90	CD57
SOX9	ITLN1	CDX2	CD44	MUC1	CD127	Cytokeratin
CD45RO	CD34	CD19	HLADR	CD4		
Multipanel						
CD127	CD21	CD138	Hoechst	CD49a	CD163	Vimentin
CD90	aSMA	SOX9	CD57	MUC1	CD15	CDX2
CD44	HLADR	CD7	CD34	CD3		
**Small intestine**						
Single panel						
CD68	CD45RO	CD4	SOX9	aSMA	Ki67	CD3
CD90	Cytokeratin	CD44	CD49a	MUC2	CD49f	Hoechst
CD31	CD38	ITLN1	CD45	CD16		
Multipanel						
SOX9	CD45RO	CD49a	CD4	aSMA	CD68	DRAQ5
CD117	CDX2	CD45	MUC2	ITLN1	CD21	Hoechst
CD66	Vimentin	CD163	CD44	CD31		

Table S3: Protein markers selected by the single and multipanel methods. Markers are listed in the order that they were selected.

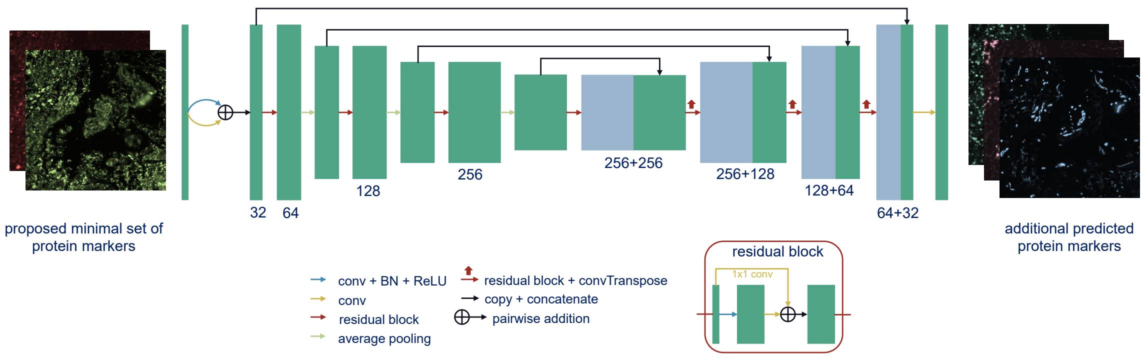

Figure S1: Architecture of the convolutional neural network used as the predictor in this paper.

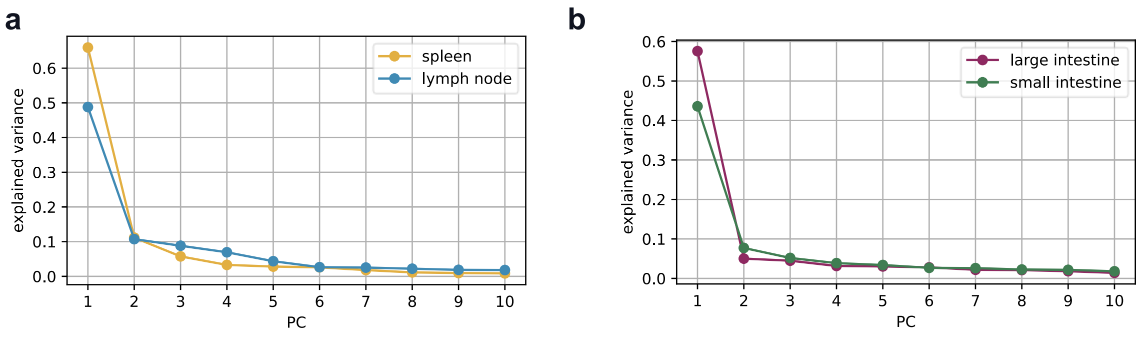

Figure S2: Variance explained by each principal component (PC) for single-cell level expression of spleen and lymph node cells (**a**) and large and small intestine cells (**b**) is shown for the test sets of the four tissues.

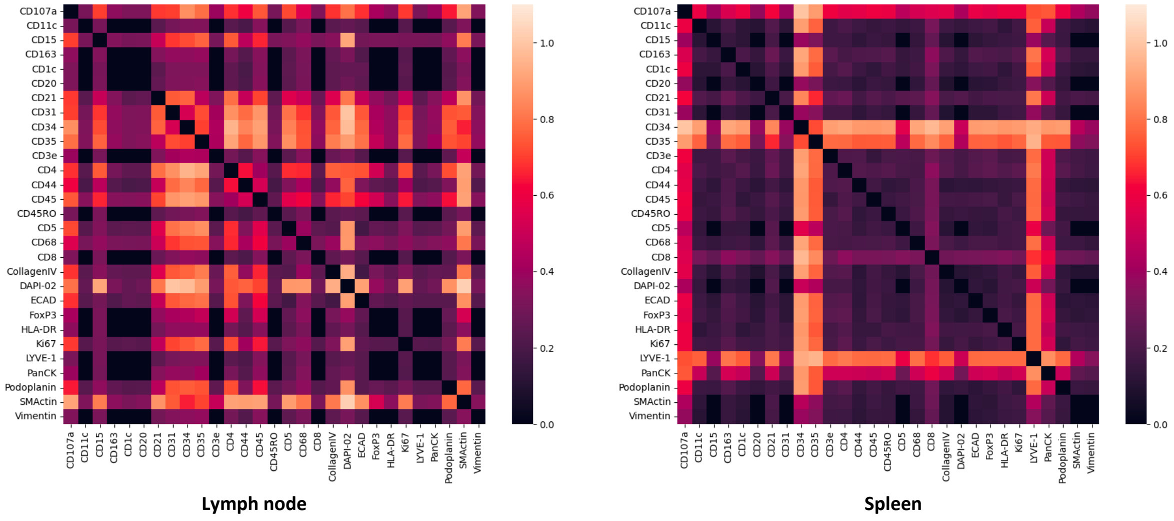

Figure S3: Heatmap showing the final graph from lymph node and spleen dataset using single panel method. Each matrix represents the final edge weights of each model after the selection process stops. Each matrix entry indicates the final weight of the edge connecting a pair of biomarkers at the corresponding coordinates. Although Figure 1 in the main text represents the graph edge bidirectionally, here we consider both directions with the same weight for the sake of simplicity, which means edges in both directions are updated simultaneously in the selection process.

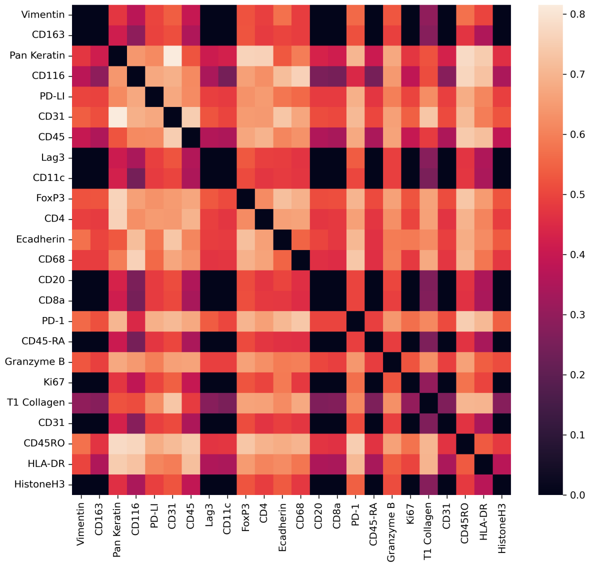

Figure S4: Similar to Figure S3, heatmap showing the final graph from the pancreas IMC dataset using single panel method.

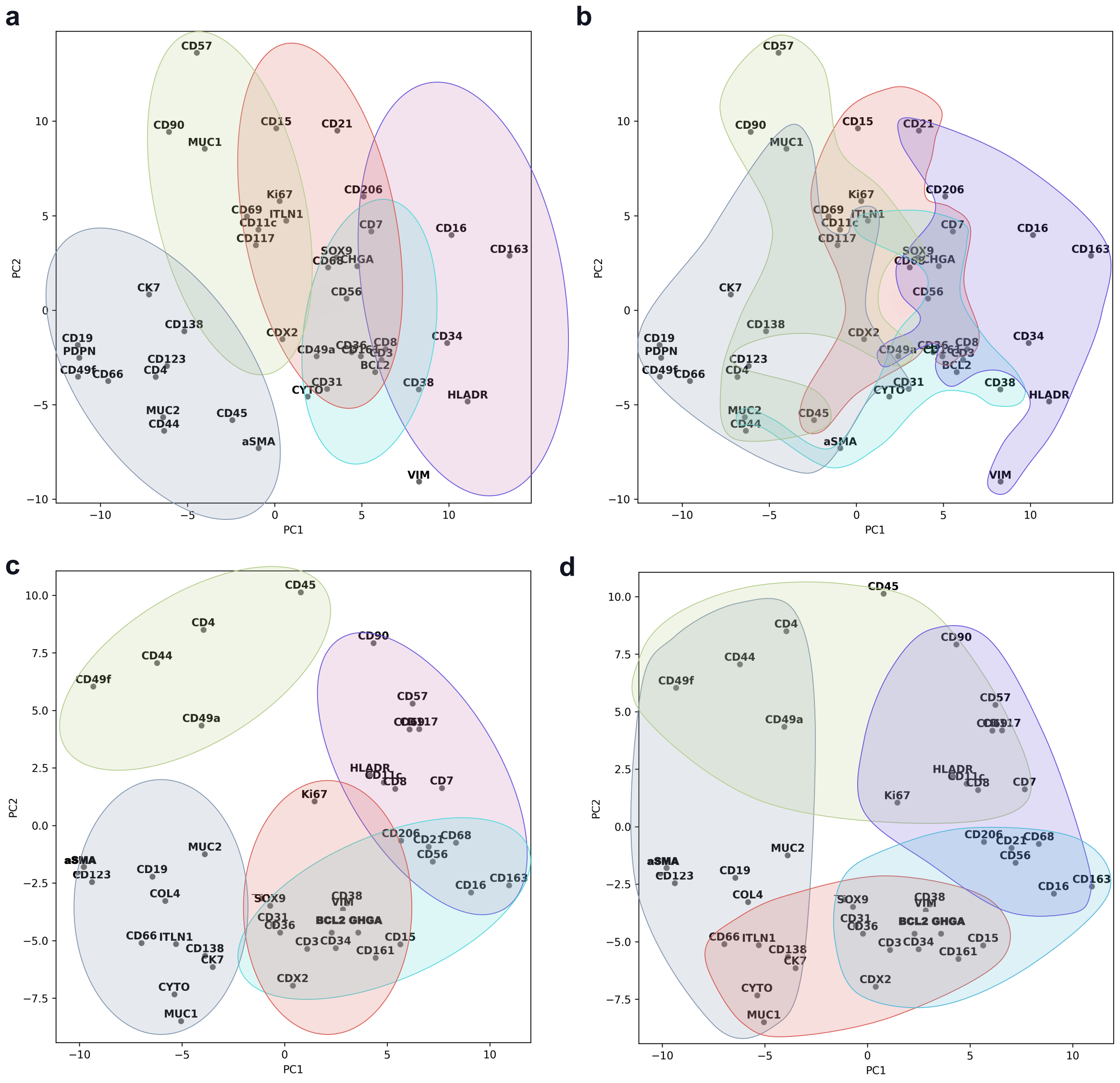

Figure S5: Clustering of markers into multiple panels. First, given a pre-defined number of panels, we identified that number of natural clusters in the dimensional-reduced PC space, **a** for the small intestine and **c** for the large intestine datasets. The blue, green, orange, cyan, and purple colored areas correspond to the initial cluster of markers into 5 panels, respectively. Then, the initialized panels were manually adjusted and extended to ensure each panel had an approximately equal number of markers and overlaps between panels, the resulting partitions are shown in **b** and **d** for the small and large intestine datasets. The colored areas correspond to the final partition of markers into 5 panels.

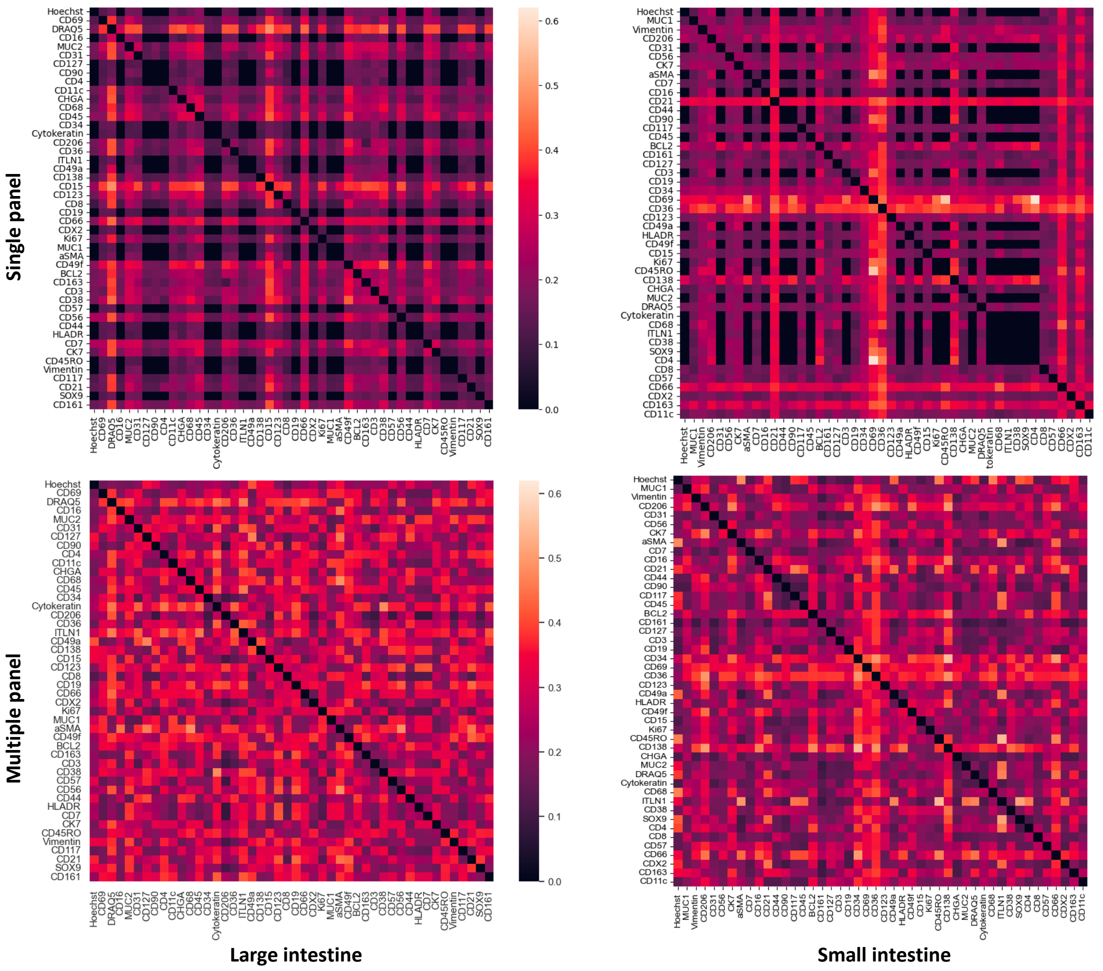

Figure S6: Similar to Figure S3, heatmap showing the final graph from the large intestine and small intestine dataset using single panel method and multi-panel method.
